# Melt Electrowriting of Graded Porous Scaffolds to Mimic the Matrix Structure of the Human Trabecular Meshwork

**DOI:** 10.1021/acsbiomaterials.2c00623

**Published:** 2022-08-19

**Authors:** Małgorzata K. Włodarczyk-Biegun, Maria Villiou, Marcus Koch, Christina Muth, Peixi Wang, Jenna Ott, Aranzazu del Campo

**Affiliations:** † INM-Leibniz Institute for New Materials, Campus D2 2, 66123 Saarbrücken, Germany; ‡ Chemistry Department, Saarland University, 66123 Saarbrücken, Germany

**Keywords:** melt electrowriting, human trabecular meshwork, glaucoma, 3D printing, poly(caprolactone), tissue engineering

## Abstract

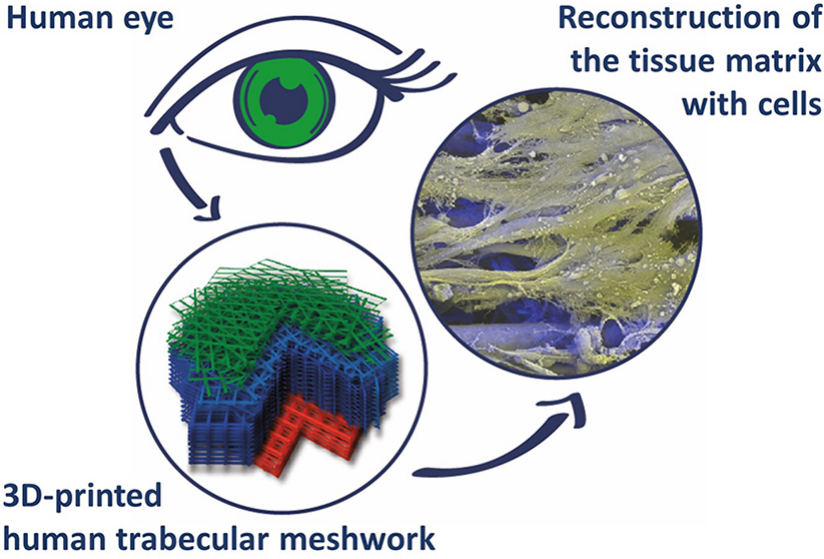

The permeability of the human trabecular meshwork (HTM) regulates eye pressure via a porosity gradient across its thickness modulated by stacked layers of matrix fibrils and cells. Changes in HTM porosity are associated with increases in intraocular pressure and the progress of diseases such as glaucoma. Engineered HTMs could help to understand the structure–function relation in natural tissues and lead to new regenerative solutions. Here, melt electrowriting (MEW) is explored as a biofabrication technique to produce fibrillar, porous scaffolds that mimic the multilayer, gradient structure of native HTM. Poly(caprolactone) constructs with a height of 125–500 μm and fiber diameters of 10–12 μm are printed. Scaffolds with a tensile modulus between 5.6 and 13 MPa and a static compression modulus in the range of 6–360 kPa are obtained by varying the scaffold design, that is, the density and orientation of the fibers and number of stacked layers. Primary HTM cells attach to the scaffolds, proliferate, and form a confluent layer within 8–14 days, depending on the scaffold design. High cell viability and cell morphology close to that in the native tissue are observed. The present work demonstrates the utility of MEW for reconstructing complex morphological features of natural tissues.

## 1 Introduction

**Figure 1:**
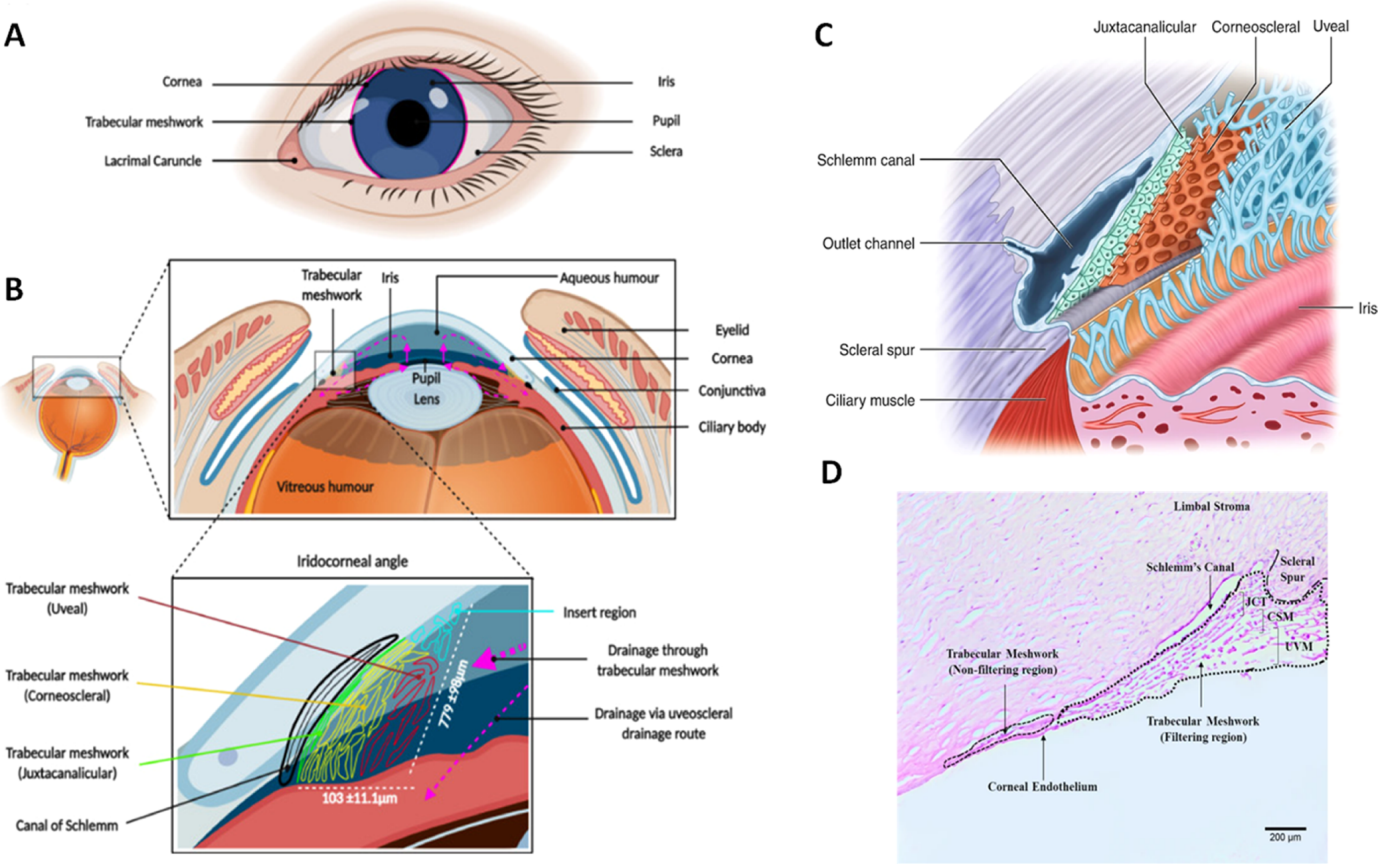
HTM. Schematics showing the location (A,B) of HTM, its triangular cross-section (bottom panel B), and the multilayer structure composed of three distinctive zones (uveal: UVM, corneoscleral: CTM, juxtacanalicular: JCT) with a characteristic porosity and beam size (B,C). Histological staining of exemplary native HTM, showing a triangular cross-section and the size of HTM (D). Reused with permission under the creative commons CC-BY-NC-ND license from ref (3), Elsevier (A,B); reused under the creative commons CC BY license from ref (4) (C) and ref (5) (D), Springer Nature.

Natural tissues and organs have multilayered structures with varying spatial gradients in morphology and/or composition that result in unique properties and functions.^1^ An example is the permeability of the human trabecular meshwork (HTM), which is achieved by a distinct porosity gradient across its thickness that regulates internal ocular pressure^2^ (see Figure 1).

The HTM is composed of three distinct zones that decrease in pore size: the uveal region (UVM) with pore sizes varying from 70 to 100 μm,^6^ the corneoscleral region (CTM) with pore sizes of 30 μm, and the juxtacanalicular region (JCT) with pore sizes of 4–7 μm^7,8^ (Figure 1C). The pores are formed by the cells and the matrix organized in lamellar beams with a thickness of 5–12 μm.^9,10^ The HTM cross-section is a triangle (Figure 1B,D) with a reported full thickness of 70–130 μm,^11^ with CTM as the main compartment, the UVM region encompassing 40.6 ± 10.0 μm,^6^ and the JCT region encompassing 2–20 μm.^6,9^ The distance from the outer layer of UVM to Schlemm’s canal can reach few hundreds of μm (Figure 1D).^5^ The resistance of HTM to fluid outflow increases across its thickness in the direction of the eye surface as the porosity decreases. Both aging and certain diseases can decrease the overall porosity in the HTM, resulting in poor regulation of the aqueous humor generation and drainage and as a consequence, increased intraocular pressure. Thus, changes in HTM porosity are a primary risk factor for glaucoma and, eventually, loss of eyesight.^7,9^ Such changes in the porosity are a consequence of alterations in the morphology and the mechanical properties of the cell–matrix layers in the HTM. Studies have shown that the elastic modulus of healthy HTM, measured locally using AFM, is ∼4 kPa, whereas in glaucomatous HTM, this value increases.^12−15^

A reconstruction of the HTM structure could help to understand how the structure and function are correlated in the natural tissue and eventually lead to regenerative solutions to associated diseases.^16^ To address this need, different scaffolds for the in vitro models of the native HTM have been reported. HTM cell cultures in microfabricated membranes of SU-8 photoresist with pores of 12 μm size were reported.^10,17,18^ The cells developed an HTM phenotype in terms of morphology, expression of HTM cell-specific markers, and ECM secretion. The proposed 2D model, although simple, was able to mimic in vivo outflow physiology: it was responsive to latrunculin-B in a dose-dependent manner,^10^ and a pathological state with increased ECM accumulation and decreased tissue permeability could be induced by treatment with steroids^18^ or with the fibrotic agent TGF-β2^17^ and counteracted by a ROCK inhibitor.^17,18^ In other studies, a functional 3D model was developed based on fibrillar hydrogels of collagen/elastin-like peptides,^14^ and the pathological state was successfully induced by dexamethasone and attenuated by ROCK inhibitor, as revealed by the increased contractility, fibronectin deposition, and hydrogel stiffening. Collagen and collagen/chondroitin sulfate scaffolds obtained via freeze-drying were also used to build in vitro 3D HTM models. Native HTM cells, after 14 days of culture, were viable and proliferated on the surface, invaded the scaffolds, and stretched along the collagen fibers.^19^ On collagen/hyaluronic acid scaffolds with different pore sizes and connectivity obtained via freeze-drying,^20^ HTM cells proliferated more in larger pores. Fibronectin expression was upregulated with increasing GAGs incorporation, and the morphology of secreted fibronectin was affected by the pore architecture, indicating the importance of the mimicry of the 3D structure. A 3D culture in Matrigel^21,22^ revealed the ability of HTM cells to adapt to chronic oxidative stress, and this was more efficient in dynamic cell culture conditions.^21,22^ The functionality of the model was corroborated by inducing pathological conditions by using TGF-β2 and dexamethasone.^22^ Despite increasing complexity, none of these studies took into account the layered structure of the HTM to recapitulate the in vivo tissue morphology. The decellularization of the native HTM without compromising its original structure was proposed.^23^ Yet, this approach is rather troublesome and, due to the very small tissue size, difficult to apply for the development of easy to handle and adjustable in vitro models. Biomimetic HTM scaffolds that could better reproduce native morphological features and help to more closely understand the structure–function relationship are still needed.^24^

Multiphasic or gradient scaffolds for in vitro engineering of tissues can be fabricated by electrospinning techniques.^25^ In this method, layers of fibrils with controlled dimensions and at controlled density can be deposited.^26^ Using melt electrowriting (MEW), a marriage between electrospinning and 3D printing, graded scaffolds with aligned fibers can be obtained. In MEW, fibers with thickness in the micrometer range are deposited from the melt with the aid of electrical voltage using a robotic stage.^27^ Well-defined, highly porous architectures can be fabricated with flexibility in the dimensions and spacing of the fibers and the number of laydown layers.^28^ MEW has been applied to print thermoplastic polymers, such as poly(caprolactone) (PCL), into structures with different pore architectures, including squared,^28−32^ rectangular,^31^ rhombus,^32^ dodecagon,^28^ triangle,^28^ or sinusoidal.^33,34^ When used as scaffolds for cell culture, cell growth has been demonstrated to depend on the fiber size^30^ and pore geometry.^35^ Multiphasic PCL scaffolds with pore sizes in the range of 250 to 750 μm and 10 μm thick fibers have been fabricated and applied for bone regeneration.^36^ PCL scaffolds with pore sizes in the range of 125 to 250 μm and fiber diameters from 4 to 25 μm were used to culture human adipose-derived stem cell spheroids.^29^ Scaffolds with a pore size of 100 to 400 μm and a fiber diameter of ca. 10 μm were used for cartilage regeneration.^37,38^ MEW has been used for skin,^39^ cardiac,^31,34^ and ligament tissue engineering^33^ and biomimetic designs of tympanic membrane^40^ and cartilage.^37,38^

In this study, MEW is applied to obtain graded scaffolds of PCL that mimic the morphological characteristics of native HTM. The methodology to prepare scaffolds containing up to 88 stacked layers of fibrils with a graded pore architecture is described. The topology, porosity, and mechanical properties of the scaffolds as a function of the design are characterized. The morphology of the primary HTM cells cultured on the scaffolds is described as a function of the scaffold’s geometry. The results indicate mechanical properties of the scaffolds matched those of natural HTM, with the cells maintaining the phenotype of native HTM cells and infiltrating the scaffolds. The utility of MEW to mimic complex morphological features of small-scale gradient natural tissues is demonstrated. This study paves the way to develop functional biomimetic high adequacy in vitro models that will allow to develop a detailed understanding of the structure–function relationship in native HTM.

## 2 Materials and Methods

### 2.1 Fabrication of MEW Scaffolds: Design and Printing Parameters

**Figure 2:**
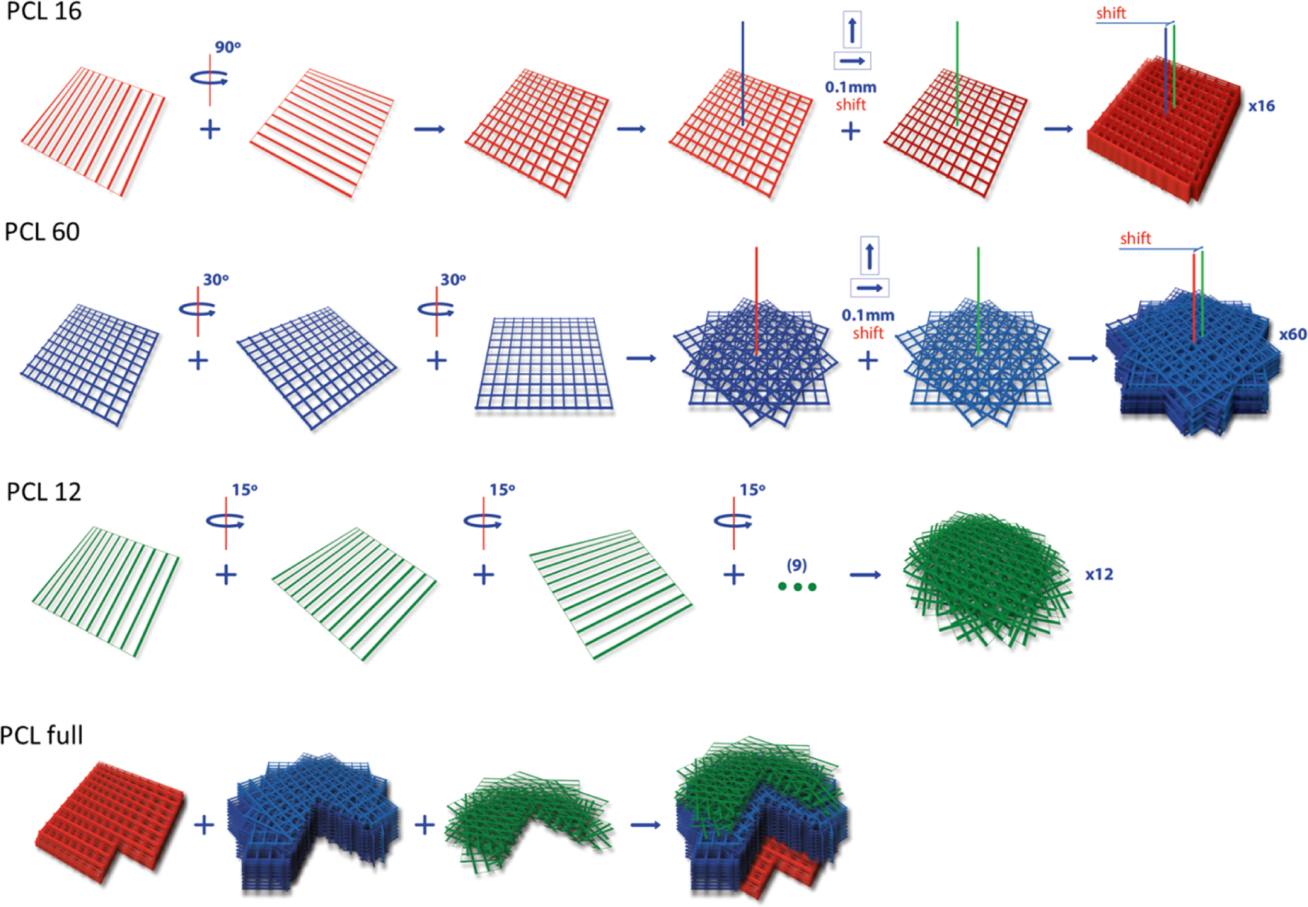
Scaffold design and rotations/displacements performed in the printing steps. PCL full structure shown with cross-section.

MEW was performed using a 3D Discovery printer (RegenHu, Switzerland) integrated into a safety cabinet (sterile conditions). The scaffold designs were programmed using BioCAD software (RegenHu, Switzerland). Four scaffold types with a different number of deposited layers (12, 16, 60, or 88), resulting thickness (125 to 500 μm), and fiber orientation between consequent layers (15, 30, or 90°) were prepared. They were named “**PCL 16**”, “**PCL 60**”, “**PCL 12**”, or “**PCL full**”, where the numbers indicate the number of deposited layers. The term “**full**” refers to the graded scaffold in which the three designs (PCL 16, PCL 60, and PCL 12) were printed consecutively (for visual presentation, see Figure 2). 25 mm × 25 mm layers were printed containing parallel strands of ca. 10–12 μm diameter. The interline spacing was set to 0.2 mm for PCL 16 and PCL 60 and to 0.1 mm for PCL 12. In PCL 16, two consecutive squares rotated by 90^o^ were printed first, followed by an 8-fold repetition of this pattern with a 0.1 mm shift in the *x* and *y* axis (16 layers in total). For PCL 12, 12 consecutive layers were printed, maintaining a rotation of 15° for each layer. For PCL 60, three consecutive squares were printed with 60° rotation each, and this pattern was repeated 20 times with a 0.1 mm shift in the *x* and *y* axis (60 layers in total). For PCL full, the three described patterns were printed consecutively in the order PCL 16, PCL 60, and PCL 12 to mimic the gradient structure of the native HTM.

Medical-grade PCL PURASORB PC 12 with *M*_n_ = 80,000 g/mol (Corbion Inc, Netherlands) was used for printing. The following printing parameters were applied: 90 °C cartridge temperature, 60 kPa pressure, 10 mm/s printing speed, 10 kV voltage, 3 mm nozzle distance from the collector, and 22G (0.4 mm) nozzle size. The material was heated to 90 °C for 2 h before the printing started and refreshed in the cartridge after every three heating cycles. Before printing, the jet was stabilized by printing a sample scaffold with the final printing parameters for 3 min. The material deposition was performed onto the collector covered with an A4 inkjet transparency film to facilitate scaffold removal. The printing process was monitored with a high-speed camera integrated with the printing head.

### 2.2 Imaging and Morphological Characterization of the Scaffolds

The printed scaffolds were imaged with a stereomicroscope SMZ800N (Nikon, Germany) using bottom illumination. The width of the printed strands was measured with integrated NISElements D (Nikon, German) software at a minimum of nine different locations in the scaffold. Four independent scaffolds of each type were printed and analyzed.

The scaffolds were also imaged by scanning electron microscopy (SEM—FEI Quanta 400 FEG). For this purpose, a sample of 5 mm × 5 mm was cut with a blade and fixed to an aluminum holder using double-sided adhesive carbon tape. For the analysis, the top view and cross-section images were taken. Secondary electron imaging was performed at 10 kV accelerating voltage in the low vacuum mode (*p* = 100 Pa water vapor, large field detector, dwell time 1.5 μs, spot size 3).

To measure scaffold porosity, the scaffold dimensions and weight were measured. The scaffold height was measured using a rheometer (as the gap value at axial force increased to ca. 0.05 N). Porosity was calculated according to the apparent density approach^41^ by the equation: *p* = (1 – *m*/*V*)/ρ_PCL_ × 100%, where *p* = porosity, *m* = scaffold mass, *V* = scaffold volume, and ρ_PCL_ = density of PCL (1.145 g cm^–3^). Measurements were performed on four samples per scaffold type. The measured porosity was between 80 and 90%. The expected porosity values were calculated for each scaffold type based on the volume occupied by printed strands (approximated as ideal cylinders) with 10 μm diameter and with the assumption that the consecutive layers are not merging but touching at one point. Consequently, based on the design the porosity was 96.07, 96.07, 92.15, and 95.54% for PCL 16, PCL 60, PCL 12, and PCL full, respectively.

### 2.3 PCL Characterization

The molecular weight of PCL as purchased, after melting and heating for 2 h and after printing, was measured by gel permeation chromatography (PSS GPC-MALLS, Germany) using the refractive index for detection. The samples were dissolved in tetrahydrofuran at 30 °C for 20 min at a concentration of 2 g/L and filtrated with a 0.45 μm polytetrafluoroethylene membrane syringe filter. 20 μL of the filtered sample was used for high-performance liquid chromatography–GPC analysis using the following conditions: 1 mL/min flow rate, 40 °C, 66 bar. For calibration, 2 g/L solutions of polystyrene standards (PSS-Polymer, Germany) were used.

Fourier transform infrared spectroscopy (FT-IR) analysis of PCL as purchased, after melting and heating for 2 h and after printing, was performed (Bruker TENSOR 27 equipped with Specac’s ATR Golden Gate, USA). Measurements were taken in triplicates; the most representative spectra are shown.

The thermal properties of the PCL [melting temperature (*T*_m_) and crystallinity] samples were measured by differential scanning calorimetry (DSC1 STAR^e^ System, Mettler Toledo). Two heating/cooling cycles at 5 °C/min were performed; reported results correspond to the second heating–cooling cycle. The crystallinity fraction was calculated from the melting enthalpy, taking 139.5 J/g as the corresponding enthalpy to 100% crystallinity.^41^

GPC, FT-IR, and DSC analyses of samples as received, after melting and printing, were performed to identify possible changes in the polymer during processing. No significant changes were observed (Table S1 and Figure S1).

### 2.4 Characterization of Wetting Properties of the Scaffolds

Contact angle measurements on the scaffolds were performed using an OCA20 (Dataphysics instruments GmbH, Germany). As a reference, a thin film of PCL obtained from the melted PCL 12 sample was used. Measurements were performed on three different scaffold samples at room temperature, using 1 μL of water droplet. In PCL full, the droplet was seeded on the side corresponding to the PCL 16 design. The images were captured using a high-speed camera. Movies of sample wetting at room temperature were captured by dipping the scaffold into poly-l-lysine solution while imaging with a stereomicroscope (Olympus SZX16) equipped with an Olympus SC50 CCD camera and Olympus Stream Basic 1.9.4 software.

### 2.5 Characterization of Mechanical Properties of the Scaffolds

Static compression and uniaxial tensile tests were performed in the wet and dry states. Wet samples were incubated for 24–48 h in phosphate-buffered saline (PBS) solution. Measurements were performed in triplicates. On the final plots representing compressive modulus data, the most representative curves are depicted. All the obtained curves are presented in the Supporting Information.

For the compression test in static mode, a TA rheometer (DHR3, TA Instruments, USA) with parallel plate geometry was used. Round samples of 6 or 8 mm radius were cut with a sharp plunger at different spots of the printed scaffolds. Prior to experiment initialization, the samples were placed at the center of the bottom plate using tweezers. For wet samples, PBS solution was added to the bottom plate around the scaffold. All experiments were performed at room temperature. Prior to measurement initialization, the upper plate of the rheometer was manually driven to get into contact with the scaffolds (ca. 0.2 N axial force), and the sample was compressed at a speed 0.2 μm/s. The initial compressive modulus was calculated from the slope of the linear part of the stress versus strain plot, in the range of 1–10% strain, using TIROS software (DHR3, TA Instruments, USA). Measurements performed below 1% strain showed high variability. One sample (out of three) measured for PCL 12 revealed the fit of linear regression *R* < 0.8 and was discharged from the calculation of the modulus value. For all other samples, the linear regression fit gave an *R* > 0.8.

Uniaxial tensile tests were performed using a Q800 DMA (TA Instruments, USA), equipped with the fixed load cell (0.0001 to 18 N range; 0.00001 N resolution). The printed scaffolds were cut as 5 mm broad stripes (25 mm long), with the longer axis parallel to fibers deposited in the first printed layer. The load was applied along the longer axis. Measurements were performed in the force range of 0.01–1 N at a constant force ramp of 0.1 N/min at room temperature and with preload force of 0.01 N. The distance between clamps was 5–6 mm. A 30 s temperature equilibration time was applied prior to the initialization of the experiment. Three samples were tested for each scaffold type to a maximum extension of 15 mm (limit of the machine). The effective elastic modulus was calculated from the slope of the linear region of the stress versus strain curve.

### 2.6 Cell Experiments

Primary HTM cells isolated from the juxtacanalicular and corneoscleral regions (P10879, Innoprot, Spain) were cultured according to the provider’s protocol. In short, the culture was set up in a 0.1% poly-l-lysine-coated T75 flasks at a seeding density of 5000 cells/cm^2^ in RPMI 1640 medium (Gibco, 61870-010) supplemented with 10% fetal bovine serum (Gibco, 10270), 1 ng/mL fibroblast growth factors, 200 mM l-glutamine, and 1% penicillin/streptomycin (Invitrogen) at 37 °C in a humidified atmosphere of 5% CO_2_. The medium was refreshed every 2–3 days. When 90% confluency was reached, the cells were subcultured.

5 mm wide stripes cut from 25 mm × 25 mm scaffolds and circular samples (6 mm diameter) cut from 6 mm × 25 mm scaffolds were used for the experiments. The samples were used in triplicates. The circular scaffolds were cut with the hollow punch tool to maintain sample integrity and minimize delamination. All scaffolds were sterilized prior to cell seeding by two immersions in 70% ethanol for 20 min, followed by washing twice with PBS and soaking over weekend in 70% ethanol. Afterward, samples were incubated in sterile PBS solution for 1.5 h at 37 °C and finally washed twice with sterile PBS.

Cells were then seeded on the four scaffold types (for scaffold PCL full, cells were seeded on the PCL 16 side). Prior to seeding, scaffolds were placed in cell culture plates: circular scaffolds in 96-well plates and the stripes in 6-well plates and fixed to the bottom of the plate by a homemade plastic ring [prepared from ThinCert (Greiner Bio-One, Germany) by removing the bottom membrane]. Scaffolds were not coated with adhesive proteins. HTM cells at passage 5 were suspended in a culture medium at 1 million/mL and seeded on the top of the scaffolds (40 μL onto small scaffolds, 50 μL onto stripes), giving the final cell density of 40000/cm^2^. After 1–2 h of incubation, an additional medium was added (200 μL per well to 96-well plate and 3 mL per well to 6-well plate). The seeded scaffolds were kept in culture for 14 days, with the cell culture medium changed every 2–3 days. Small scaffolds were used for the cell metabolic activity test, performed with an alamarBlue assay. 5 mm-width stripes at day 1, day 8, and day 14 were cut with a sterile blade to be used for viability assay, immunostaining, and SEM investigation or further culture. Nonadherent plates were used throughout the study, besides the controls with cells intentionally cultured on plastic, to minimize the influence of the cell growth on the bottom of the well plates on the experimental results. Additionally, prior to the alamarBlue assay and staining (live/dead, immunostaining), samples were moved to fresh wells to study solely the cells growing on the scaffolds.

#### 2.6.1 Cell Viability

A live/dead cell viability assay was performed following the manufacturer’s protocol (Sigma-Aldrich). Briefly, PBS solutions of 20 μg/mL propidium iodide, to stain dead cells in red (excitation/emission ≈ 535/617 nm), and 6 μg/mL fluorescein diacetate, to stain live cells in green (excitation/emission ≈ 490/515 nm), were prepared. Samples in triplicates on day 1, day 8, and day 14 were removed from the medium, placed in a fresh well plate, and incubated with 100–250 μL of staining solution for 10 min. After 2× washing with PBS, cells on the scaffolds were imaged with a PolScope fluorescence microscope (Zeiss, Germany). Three images per sample were used for analysis (besides four exceptions, where two images were analyzed and one exception with one image). Images were analyzed with ImageJ software. After brightness and contrast adjustment, cell counting was performed with the function “find maxima.” The cell viability was calculated using the following equation: % viability = (no. of live cells/total no. of cells) × 100.

#### 2.6.2 Cell Metabolic Activity, Nuclei Shape, and Scaffold Infiltration

Cell metabolic activity was quantified using the alamarBlue assay (Invitrogen). Samples on days 1, 4, 8, 11, and 14 were transferred to a fresh well plate, and the alamarBlue reagent was added in a 1:10 ratio to the culturing medium. After 3.5 h, the scaffolds were removed, and the absorbance of the medium at 570 nm was analyzed with a multidetection microplate reader Synergy HT (BioTek Instruments; Vermont, USA). For each scaffold type, three independent samples were analyzed. Results were normalized to the control (cells seeded on the bottom of 96-well plate at day 0 at the density of 40000/cm^2^).

On day 14, samples in triplicates were fixed with paraformaldehyde (PFA) 4% w/v for 10 min, followed by three washes with PBS. After permeabilization with 0.5% w/v Triton-X 100 (TX) for 10 min and PBS wash, cell nuclei were stained with 1:1000 DAPI (4′,6-diamidino-2-phenylindole, dihydrochloride, Thermo Fisher) in PBS and washed with PBS again. Imaging was performed with Nikon Ti-Eclipse (Nikon Instruments Europe B.V., Germany) with a Sola SE 365 II (Lumencor Inc., Beaverton, USA) solid-state illumination device and an Andor Clara CCD camera. Three independent samples imaged at 20× magnification were used to analyze nuclei parameters: length and width and the ratio of length to width further denoted as AR (aspect ratio). The pictures were processed with ImageJ software by color threshold adjustment, followed by watershed processing and the use of the “analyze particles” function (size limit 50–150 μm). Scaffold infiltration by cells was investigated using a confocal microscope (Zeiss LSM 880) and imaging in the *z*-stack mode.

#### 2.6.3 Immunostaining and Fluorescence Microscopy

Immunostaining of the scaffolds for F-actin cytoskeleton and αβ-crystallin, an HTM characteristic marker, was carried out on day 14. Pieces of the scaffolds were fixed in cold 4% PFA in PBS solution for 10 min, followed by 2–3 times rinsing in PBS for 5–10 min and stored at 4 °C till staining.

Prior to staining, samples in triplicates were permeabilized with 0.5% Triton-X 100 in PBS for 15 min and blocked with 0.1% Triton-X 100 in PBS mixed with 5% w/v BSA (denoted further as PBST solution) for 20 min.

Afterward, scaffolds were incubated in 1:200 Alexa Fluor-488 Phalloidin (Thermo Fisher) for cytoskeleton staining and in 1:500 anti-αβ-crystallin (Abcam, Cambridge, MA) in PBST for 1 h at room temperature. Samples were subsequently rinsed with PBST (three times) and incubated with secondary antibody Alexa Flour-594 goat antimouse (Thermo Fisher, 1:100 dilution) for αβ-crystallin detection. Finally, samples were rinsed with PBST (2×), incubated with 1:1000 DAPI (Thermo Fisher) in PBS for 20 min for nuclei staining, and rinsed in PBS (2×). Imaging was performed using Nikon Ti-Eclipse (Nikon Instruments Europe B.V., Germany) with a Sola SE 365 II (Lumencor Inc., Beaverton, USA) solid-state illumination device and an Andor Clara CCD camera for detection.

#### 2.6.4 SEM of Cell-Loaded Scaffolds

Similar imaging conditions, as described above for scaffolds without cells, were used. The scaffolds with cells, one per type, were fixed using 2% glutaraldehyde in phosphate buffer, dehydrated, and dried using a graded series of increasing water/ethanol mixtures and hexamethyldisilazane (HMDS) before imaging. The scaffolds were incubated for 10 min in 50% v/v ethanol–water, 10 min in 70% v/v ethanol–water, 10 min in 80% v/v ethanol–water, 10 min in 90% v/v ethanol–water, 10 min in 96% v/v ethanol–water, 2 × 20 min in 100% ethanol, 10 min in 50% v/v ethanol–HMDS, and 10 min in 100% HMDS and dried under ambient conditions overnight.

### 2.7 Statistical Analysis

All the results are reported as the mean ± standard deviation. Statistical differences were analyzed using one-way analysis of variance (ANOVA) and posthoc Tukey test or unpaired *t*-test, performed with In Stat3 software. Differences with *p* < 0.05 were considered significant.

## 3 Results and Discussion

### 3.1 Scaffold Design and Printing

Inspired by the distinctive structure and pore size of the consecutive layers of the native HTM, three different scaffolds were designed (PCL 16, PCL 60, and PCL 12; the numbers indicate the number of deposited layers). The targeted dimensions for printing were a fiber diameter of 10 μm, recapitulating the typical trabecular beam size, and 80% porosity, with effective pore sizes decreasing in the order PCL 12 < PCL 60 ≤ PCL 16. Scaffolds PCL 16 and PCL 60 (Figure 2), containing 16 and 60 printed layers, respectively, were constructed by a periodically repeated square mesh pattern (with 200 μm interfiber spacing). In PCL 16, the consecutive square meshes were shifted by 1/2 of the period (100 μm) in the *x* and *y* axis; in PCL 60, the consecutive square meshes were rotated by 30°, and after every 3 square meshes, a shift of 1/2 of the period (100 μm) in the *x* and *y* axis was applied. In PCL 12 (Figure 2), with 12 printed layers, fibers in each layer were printed with a smaller interfiber distance (100 μm) and were rotated by 15° to achieve smaller pore sizes than in previous designs. A PCL full scaffold was also fabricated (Figure 2) by superposing the three previous designs to reconstruct the multilayer HTM structure.

**Figure 3:**
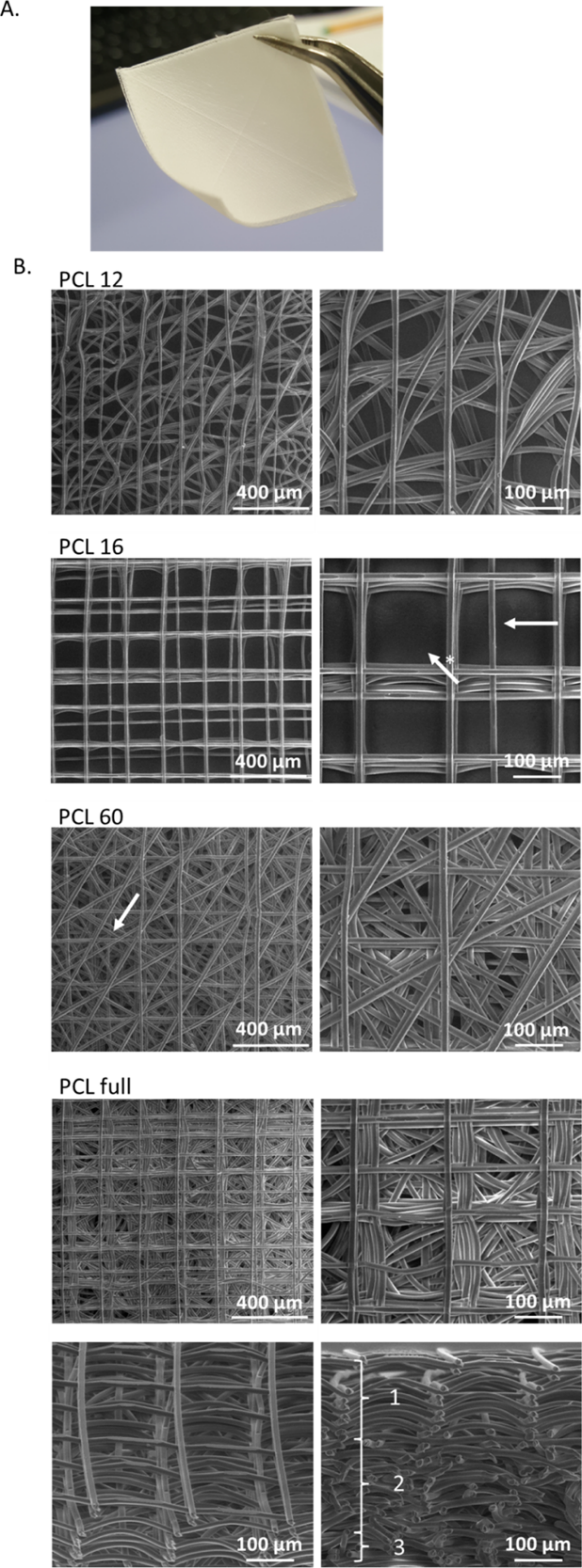
Images of printed scaffolds. Macroscopic view of the printed PCL full scaffold (A). SEM top view images at 200× (left) and 500× (right) magnification. A few structural defects are highlighted: white arrows show an example of imprecisely positioned fibers, and the white arrow with (*) shows a missing vertical fiber according to the design. The bottom row presents the tilted and cross-section view of PCL full at magnification 500×. Three distinctive layers are marked in white (B).

Medical grade PCL ca. 100,000 kDa (see Table S1) was selected as electrowriting ink due to its good processability^42^ and biocompatibility.^43^ Printing parameters were optimized to achieve the best shape fidelity, besides PCL 12 design, as discussed later. Scaffolds contained superposed layers of 25 mm × 25 mm area (Figure 3A) and were printed layer-by-layer. The layers contained aligned fibers with a diameter of 10–12 μm (Table 1) and spacing of 200 μm (PCL 16 and 60) or 100 μm (PCL 12). 200 μm was the minimum interfiber distance that we could achieve with high fidelity, in agreement with previous work on MEW with PCL by other authors.^33^ Shorter interfiber distances lead to lower precision in the fiber deposition due to the charge built up at the collector plate and the consequent alterations in the electrical field.^44^ PCL 16 and PCL 60 scaffolds displayed straight and parallel fibers, albeit with a few distortions in the printed fibers (indicated by white arrows in Figure 3B). In PCL 12, in order to reduce the pore size, a smaller interlayer rotation (15°) was used, and a 100 μm interfiber distance was attempted, though this was at the cost of a more irregular material flow and bending of the deposited fibers. Additionally, printing below the critical translational speed was used intentionally to introduce fiber crimps and further decrease pore sizes.

**Table 1:** Features of Scaffolds Printed with Different Designs: Height, Fiber Diameter, Theoretical and Measured Porosity, and Water Contact Angle

			porosity (%)			compression modulus *E* (kPa)		tensile modulus *E* (MPa)	
	scaffold height (μm)	fiber diameter (μm)	theoretical	measured	contact angle (deg)	dry	wet	dry	wet
PCL 12	125 ± 10	10.0 ± 0.6	92.15	86.8 ± 1.9	130 ± 2	11.2 ± 3.3	5.6 ± 1.9	13.0 ± 1.7	11.1 ± 1.1
PCL 16	140 ± 19	11.8 ± 1.4	96.07	91.2 ± 1.6^a^	130 ± 4	63.8 ± 79.9	66.4 ± 87.6	7.2 ± 2.1	5.6 ± 1.8
PCL 60	299 ± 20	10.2 ± 0.8	96.07	84.7 ± 0.4	126 ± 1	87.9 ± 75.6	216.4 ± 175.7	7.6 ± 0.9	7.3 ± 1.0
PCL full	506 ± 18	11.9 ± 1.3	95.54	84.2 ± 1.5	126 ± 8	358 ± 235	35 ± 17^b^	6.9 ± 1.1	6.7 ± 1.4

a Statistically different from all other scaffolds.

b Statistically different from the dry scaffold.

The thickness of the printed scaffolds (Table 1) varied between ca. 125 μm (PCL 12) and ca. 500 μm (PCL full). The total thickness of scaffolds was smaller than the sum of the consecutive printed layers due to the layers merging, assuring proper connectivity and minimizing delamination. The measured porosity, based on the previously reported apparent density approach,^45^ was 84–91%, in the order of PCL full ≈ PCL 60 < PCL 12 < PCL 16 (see Table 1), whereas the theoretical porosity, calculated for ideal scaffolds with 10 μm diameter straight strands and neglected merging between consecutive printed layers, was calculated as 92–96%. The measured porosity was smaller than the theoretical one for all the scaffolds due to the merging between layers, resulting in material densification, nonideally straight strands printing for PCL 12, and a fiber diameter exceeding 10 μm in the case of PCL 16 and PCL full scaffolds (Table 1). As a result, more material per unit of the scaffold volume was deposited while printing than that calculated theoretically. The smallest difference between theoretical and measured porosity was detected for PCL 16 scaffolds, indicating the highest printing fidelity for this design. On the contrary, the highest deviation between theoretical and measured values was observed for PCL 60, which we assigned to the most pronounced merging of the consecutive printed layers (increased material densification) due to the highest number of layers, resulting in the highest scaffold weight. The porosity % measured for the PCL 12 and PCL 60 was in the same range. The distance between the strands belonging to the same layer was smaller for PCL 12, yet the higher number of layers in PCL 60 and increased strands merging compensated for this effect.

### 3.2 Wetting Properties and Stability of the Printed Scaffolds in Watery Media

The wetting properties of the scaffold were studied by measuring the water contact angle. Values between 125 and 130° were observed (Table 1), indicating hydrophobicity of the scaffolds as a consequence of their surface structure. As a reference, the PCL film had a contact angle of 73.2° ± 2.1°. Importantly, the scaffolds immersed in an aqueous solution were wetted instantaneously in the case of PCL 12 and PCL 16 and slower in the case of thicker structures (PCL 60, PCL full). The facilitated wetting of PCL 12 and PCL 16 can be explained by easier removal of the air pockets in thinner scaffolds during immersion.^46^

All scaffolds remained stable during immersion in a cell culture medium for 14 days. No delamination or disintegration was observed. This result indicates good interfibrillar adhesion between the printed fiber and the one below. Note that the degradation time of PCL in water is 12 months.^47^

### 3.3 Mechanical Properties

**Figure 4:**
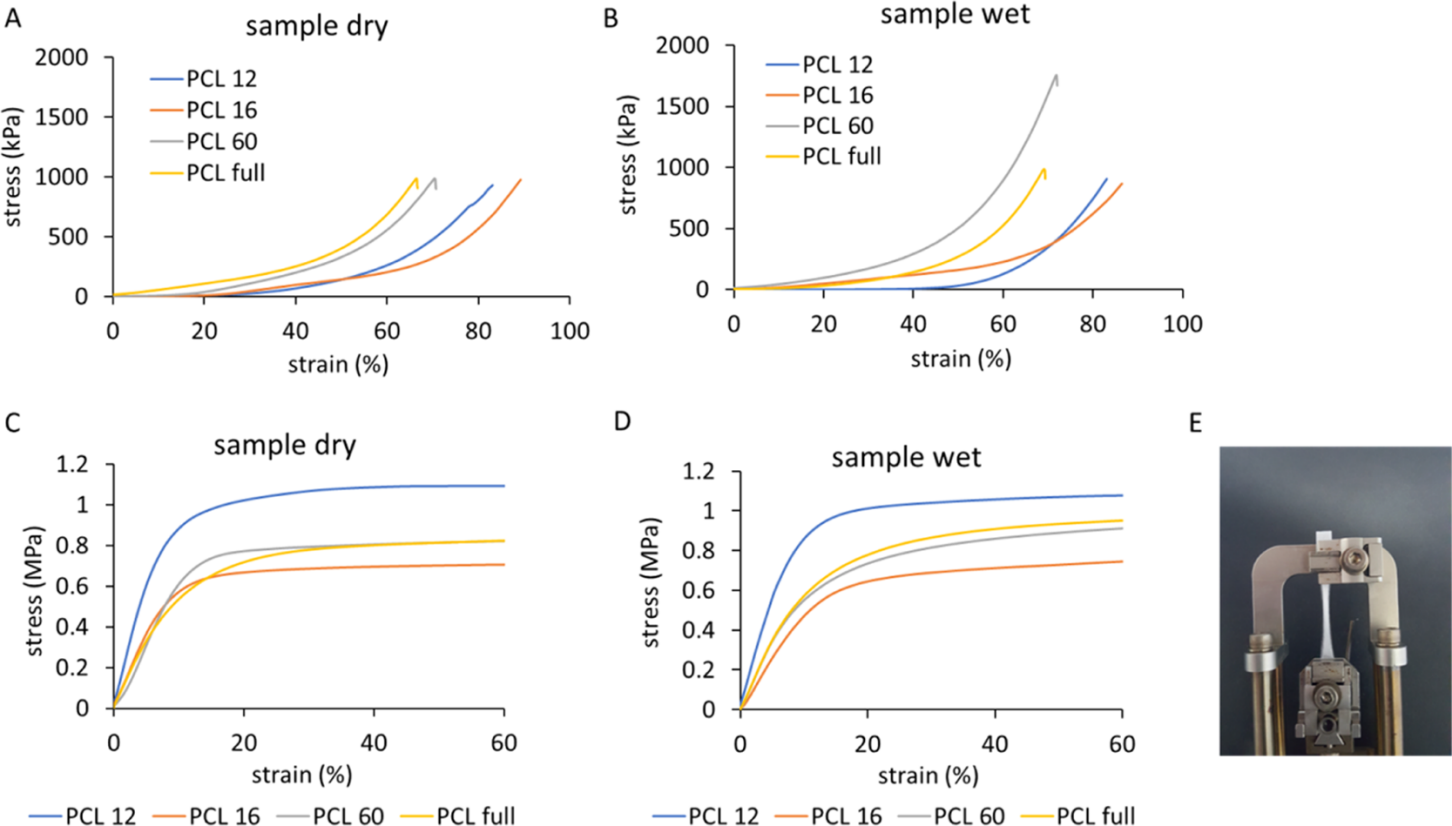
Mechanical testing of different scaffolds. Stress–strain curves of the scaffolds obtained in a static compression test of dry (A) and wet (B) samples and a tensile test of dry (C) and wet (D) samples. PCL 60 sample at the end of the test after ca. 300% elongation (E).

Uniaxial compression tests of the scaffolds in the static mode were performed. The strain–stress curves (Figures 4A,B and S2) show two regions of different slopes: an initial region of lower slope at strains <40% (PCL 60 and PCL full) and <60% (PCL 12 and PCL 16), which was followed by a densification region with a higher slope.^48^ Based on the strain–stress curves, the compression behavior of PCL full seems to be dominated by the compression behavior of the PCL 60 region, which is the main constituent related to the scaffold volume (60 layers of 88 layers in total). The elastic modulus of the printed scaffolds was extracted from the initial slope of the curve (<10% strain). The obtained moduli are in the range of 6–360 kPa (see Table 1), with the values showing the trend: PCL 12 < PCL 16 < PCL 60 < PCL full for the dry samples (no statistical significance). This subtle effect correlates to the scaffold porosity (PCL full ≈ PCL 60 < PCL 16 < PCL 12) and the number of printed layers (PCL 12 < PCL 16 < PCL 60 < PCL full) and could be explained by more extensive merging between the layers leading to an increased connection at the nodes. The compressive modulus values show a similar trend to previous studies on highly porous PCL scaffolds, suggesting that the modulus is related more to porosity and less to fiber alignment or design geometry.^48^ However, PLC 16, in the dry and wet states, seems to reach the highest strain before failure, which could be assigned to the geometrical arrangement of the strands, specifically to the support given by the overlapping layers (strands in the alternating layers are printed at the same *xy* position). The compression measurements were performed in the dry and wet states (note that PCL shows limited swelling when immersed in water^49^). There were no statistical differences between wet and dry samples, except for PCL full, which revealed a decreased modulus value after 24–48 h of incubation in PBS at RT. It was shown earlier that with an increasing scaffold height, the printing accuracy decreases due to the residual charges accumulated in already printed strands, causing material repealing and disordering.^50^ Less accurate fiber placement and material repealing while printing could influence the cooling rate of deposited fibers, weakening the connection between consecutive layers, and consequently, the integrity of the scaffold after immersing in PBS. Disconnected layers could slide over each other, decreasing the overlap of the printed strands and final scaffold stiffness. Note, that these disconnections, especially if not very extensive, may have only negligible influence on the results of the tensile measurements as the scaffold’s layers are kept in the initial relative positions in the testing direction by the device’s clamps.

The compression moduli values, although characterized by significant variability, are in agreement with previous reports. For multilayered PCL scaffolds printed with MEW with a square-based design, ca. 20 μm fiber diameter, 200 μm interfiber spacing, and 1 mm height, compression moduli of ca. 14 kPa^51^ or slightly below 1 MPa^37^ were reported. The ranges of compression moduli obtained in the study are close to those of collagen-based scaffolds used for culturing HTM cells by other authors (7kPa^19^). The compression modulus of HTM in healthy and glaucoma HTM has been reported to be 4 and 80 kPa, respectively, yet measured by AFM.^13^ Other studies reported only 1.4-fold increase in the storage modulus of glaucomatous HTM.^15^ The scaffolds withstood strains up to at least 60% before failure (Figure S3). For PCL 12 and PCL 16, the failure was not recorded due to the measurement limitation for thin samples. The failure behavior in PCL full was dominated by the thickest PCL 60 layer.

The stress–strain curves in the uniaxial tensile test of the scaffolds are shown in Figures 4C,D, S4, and S5. An initial linear region with a steep slope was followed by a long plateau region. No breaking point was reached below 300% elongation, which was the extension limit of our equipment (Figures 4E and S5). The tensile modulus was in the range of 5.6–13 MPa for all the designs (see Table 1). These values are in agreement with those previously observed for square-based scaffolds,^31,37^ where the range of a few MPa was reached. The moduli of PCL 16, PCL 60, and PCL full was similar; the slightly higher modulus of PCL 12 might be associated with lower porosity (the highest material volume fraction). In PCL 16, fibers elongated in the stretching direction without a visible fiber break due to the alignment in the design, whereas in PCL 12, PCL 60, and PCL full, failure of consecutive, single fibers was observed during the experiment (Figure S5C). No variation in the tensile modulus was observed with the humidity of the sample.

Different values for Young’s modulus of dissected HTM have been reported. Human HTM segments measured by the uniaxial tensile test showed Young’s modulus of 515 ± 136 kPa, while porcine HTM showed 25 kPa.^52^ In a different report, 12.5 MPa was measured for glaucomatous human HTM and 42.6 MPa for normal tissue.^53^ The results obtained here seem to be within the range relevant for physiological studies.

### 3.4 Cell Culture Studies

**Figure 5:**
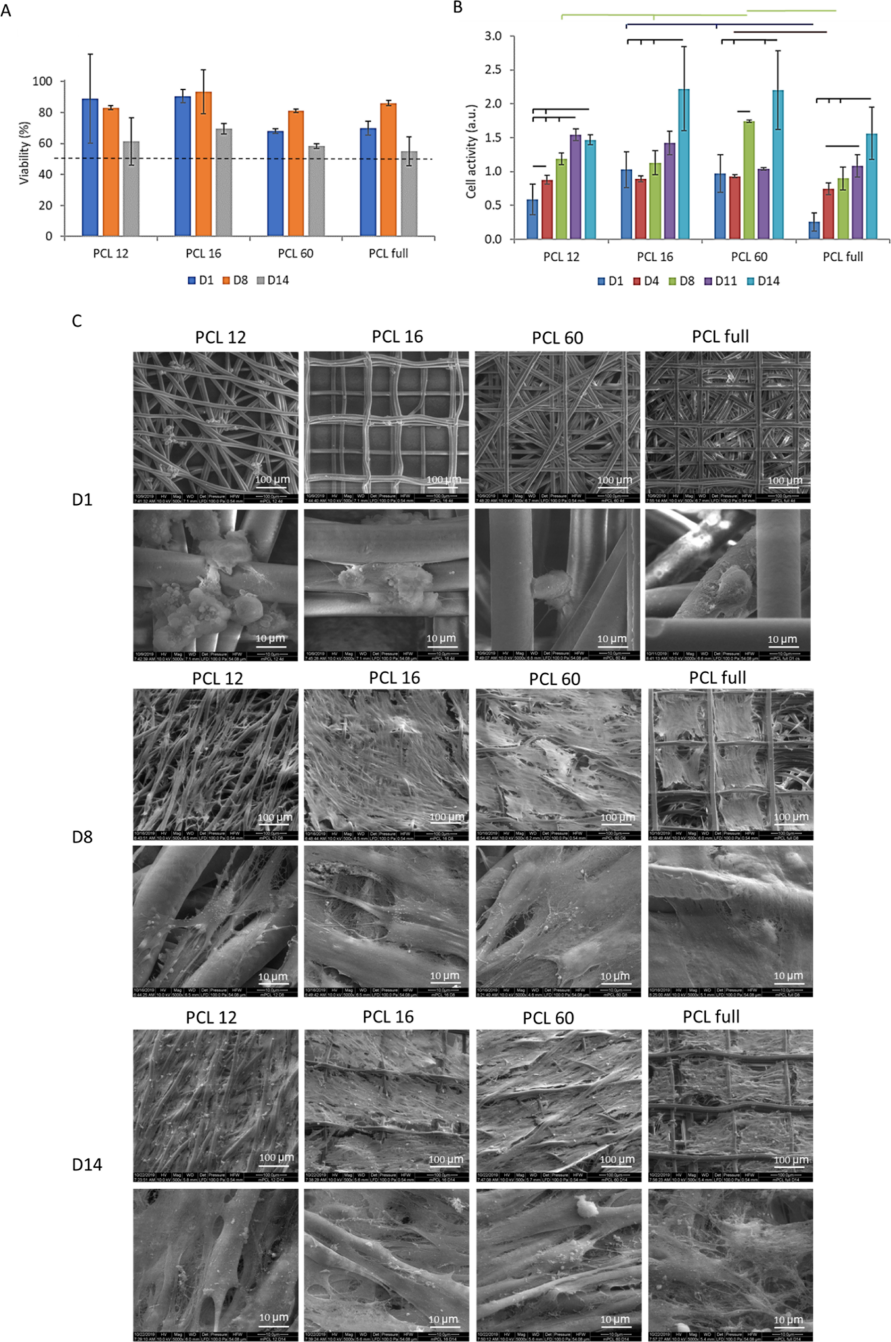
Biological evaluation of the cells growing on different scaffolds. (A) Viability of the cells based on live/dead assay staining. Threshold of 50% viability marked by the broken line. (B) Cell metabolic activity based on the alamarBlue assay. (C) SEM images of scaffolds with cells on day 1, day 8, and day 14 at 2 magnifications: 500× (top; scale bars: 100 μm) and 5000× (bottom; scale bars: 10 μm).

The ability of the printed scaffolds to support the culture of primary HTM cells was tested in viability and metabolic activity tests. HTM cells were seeded on the scaffolds. We performed a live/dead assay to assess cell viability, followed by fluorescence imaging. In PCL 12 and PCL 16 samples, the stained cells were visible through the scaffolds, whereas for the PCL 60 and PCL full scaffolds that contain a high number of layers, only cells in the more superficial layers were available for examination and included in the analysis. The cell viability varied between 60 and 90% at day 1, day 8, and day 14 after seeding (Figure 5A). PCL 60 and PCL full show a similar trend, which can be explained by the fact that PCL 60 constitutes the main part of PCL full and that the cells after seeding could penetrate the PCL 60 layer. A relatively lower viability was detected in those scaffolds compared to PCL12 and PCL 16, which might be due to a more hindered diffusion of the medium inside more dense and thicker constructs (PCL 60 and PCL full), causing cell death in deeper layers still visible under optical investigation. The drop in viability for all the samples on day 14 is attributed to the high cell density achieved at that time point. The large error in the measurements is due to imaging difficulties as a consequence of the reflection of light by the scaffold.

Cell metabolic activity within the substrates was quantified by the alamarBlue assay (Figure 5B). Metabolic activity was observed across all scaffolds, and it increased with culture time, which we assigned to increased cell proliferation, regardless of the design. Yet, it should be noted that the increase in the readout could be also caused by increased metabolic activity of the cells and not directly their proliferation. The profiles observed for PCL 16 and PCL 60 are characterized by unexpected data fluctuation and deviation from continuous increase of metabolic activity in time. One of the possible reasons could be the outgrowth of the cells from the scaffolds to the culturing well plate. Before the alamarBlue test, the samples were transferred to a new well for the measurement, and it is possible that the cells bridging the scaffolds and well plate in some cases stayed attached to the scaffolds and, in others, to the well plate.

#### 3.4.1 Cell Morphology and Distribution on the Scaffolds

The morphology of HTM cells on the scaffolds on days 1, 8, and 14 after seeding was studied by SEM. On day 1 (Figure 5C), cells accumulated at the nodes or fiber crossings of the scaffolds and had a rounded morphology. On day 8, cells spread out on the scaffolds, revealing a typical spindle-like shape (native HTM cell morphology) and overlapping processes.^19^ However, different morphologies and coverage areas were observed depending on the scaffold design. Cells on PCL 16, PCL 60, and PCL full (which was seeded on the PCL 16 surface) spread out in all directions and spanned the fibers of the scaffold’s surface, filling the interfiber space, and formed a dense cell layer. The covered area by the cells was larger in PCL 16 and 60 than in PCL full. In contrast, cells on PCL 12 scaffolds elongated along the fibers and, in some cases, bridged adjacent fibers but did not form continuous cell layers of an appreciable area. On day 14, PCL 12 and PCL 60 scaffolds were covered by a uniform and dense cell layer, whereas the cell layer on PCL 16 and PCL full had some empty areas (see Figure S6). The SEM investigation (see Figures S7 and S8) and the nuclei distribution tracked with confocal microscopy (Figure S9) confirmed that cells could infiltrate all the scaffolds. The differences in cell densities inside the scaffold observed by fluorescence microscopy is caused by adsorption of light due to the PCL mesh that blocks light and leads to an underestimation of cells in the center of thick scaffolds. The most uniform infiltration was detected for thinner scaffolds (PCL 12 and PCL 16). The smaller pore sizes facilitated bridging of the fibers at earlier time points and confluent layer formation on the surface of the scaffold. For planar SU-8 scaffolds reported before, it was observed that the cells have difficulty growing on pore sizes bigger than 15 μm.^18^ For those scaffolds^18^ and 3D hydrogel-based scaffolds produced by freeze-drying,^19^ limited cell penetration into the pores was observed, whereas in our models, the cell infiltration was significant. In the follow-up study, the authors have shown that the cells seeded on freeze-dried hydrogel-based scaffolds with large pore sizes (in the range of 200 μm) proliferate more than on the samples with small pores (in the range of 20 μm), most probably thanks to the higher surface area available for cells growth and easier nutrient and oxygen transport. The nonaligned pores were also more beneficial for cell growth than the aligned ones due to the alternative routes for cell proliferation and migration.^20^

#### 3.4.2 Cellular Identity/Maintenance of the Phenotype

HTM cells in the natural tissue have elongated cell shapes bridging multiple adjacent fibers and elongated nuclei and reveal specific markers.^10^ To further analyze the phenotype of HTM cells within the scaffolds, cells were stained with DAPI, phalloidin, and anti-αβ-crystallin to reveal nucleus elongation, the disposition of actin fibers, and expression of the HTM cell-specific marker, respectively.

**Figure 6:**
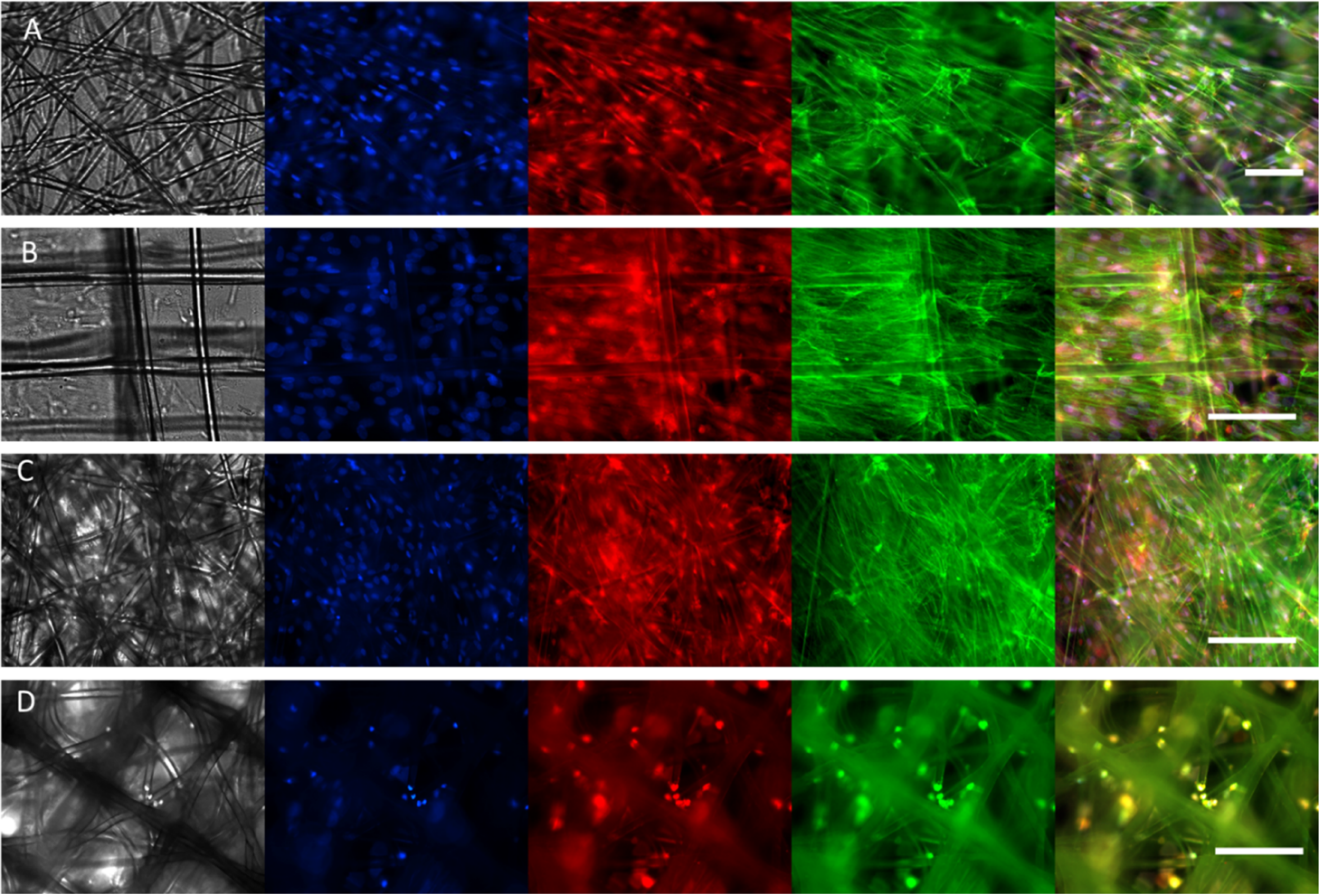
Fluorescence images of the cells cultured for 14 days on different scaffold types: PCL 12 (A), PCL 16 (B), PCL 60 (C), PCL full (D). The first channel represents bright-field (in gray), the second channel, DAPI nuclei staining (blue), the third channel, αβ-crystallin (red), the fourth channel, phalloidin actin staining (green), and the last tile is a merged representation. Scale bars: 100 μm. Note that due to light reflection, the light imaging of the cells in multilayer scaffolds is impeded.

The nucleus shape in cells on the different designs based on DAPI staining was estimated after 14 days of culture. The AR parameter was calculated as a ratio of nucleus length to width (an example of image preparation for quantifications is provided in the Supporting Information, Figure S10). The nuclei of the cells were elongated (AR ≥ 1.5: 1.7 ± 0.4 for PCL 12, 1.7 ± 0.5 for PCL 16, and 1.5 ± 0.4 for PCL 60, see also Figure 6) with greater elongation visible for PCL 12 and PCL 16. The native HTM cells in the in vivo conditions typically reveal elongated nuclei.^10^ The AR reported in previous studies was 1.1 for 2D culture on a porous membrane (insert) and 2.0 for an optimized SU-8 photoresist membrane with pores of 12 μm.

Actin staining after 14 days of culture showed elongated structures in the cells cultured on all the scaffolds, besides PCL full (Figures 6 and S11), indicating that cells are attached to the scaffold and are able to build cytoskeletal actin fibers. Owing to the reflection of light by the thick, nontransparent scaffold, the cellular cytoskeleton and spreading of the cells on PCL full was difficult to observe based on actin staining, and the clear alignment observed in other studies^10^ was difficult to prove. Nevertheless, SEM images indicate the presence of stretched cells, bridging adjacent fibers in all printed designs. αβ-Crystallin was expressed by HTM cells on all the scaffolds. αβ-Crystallin is a characteristic marker of the JCT region in HTM^9^ and is not expressed in conventional 2D cultures.^18^ Therefore, we concluded that the MEW-printed 3D HTM model revealed improved properties, leading to the appearance of the key in vivo-like HTM characteristics.

The obtained data show that the printed PCL scaffolds were suitable for the culture of HTM cells and maintained the native phenotype of JCT cells, including the spreading and formation of cell layers, with cell–cell interfaces between adjacent cells. The native JCT is covered by two to five discontinuous cell layers, with the cells making a satellite connection with the endothelial cells lining the Schlemm’s canal.^7^ The cells in native UVM and CTM regions have more rounded shapes, whereas cells in the JCT region adopt elongated shapes.^2,24^ In our scaffolds, the JCT phenotype seems to be reconstituted. Changes in the printed designs, such as more open porosity (less dense structure in the z direction) and a smaller number of the printed layers in the middle zone, might allow reconstruction of the phenotype of HTM cells in the UVM or CTM layers.

In future work, to achieve a closer mimic of native HTM, the altered design of the middle zone could be proposed, providing a thinner structure with smaller pores. This is relatively challenging to obtain using commercial MEW printers, yet there are some reports on the MEW scaffolds with the interfiber distance reduced to 40 μm.^54^ To analyze the scaffold functionality, the permeability tests at the physiological pressure and analysis of cellular response to the drugs typically used in glaucoma disease will be performed. The proposed scaffolds, with future adjustments, are envisioned for high accuracy for in vitro disease and drug testing models. Owing to the very small size of the tissue, the technological limitation makes it challenging to use the scaffolds as patient implants in the near future.

## 4 Conclusions

This study demonstrates the feasibility of in vitro reconstruction of some features of the HTM using MEW scaffolds of PCL, that is, graded porosity and native trabecular beam size. Scaffolds with three different designs were printed in an effort to build the three distinctive matrix layers of native HTM and one combined design containing a stacking of the three layers with a layered morphology and porosity. Printed scaffolds having relevant mechanical properties were stable (no delamination effect) during 14 days of cell culture and supported 14 days of culture of primary HTM cells. Scaffold design influenced cell morphology: the thinner scaffolds showed better cell infiltration, and the smaller pores sizes facilitated cell elongation along the fibers; the bridging of multiple adjacent fibers at an earlier time point (8 days) and confluent and more uniform layer formation on the surface of the scaffold at the later time point (after 14 days). HTM cells on the scaffolds showed elongated nuclei and a well-developed actin cytoskeleton and revealed a specific marker observed only in 3D culturing conditions, which are features characteristic of HTM cells in the natural JCL layer of the native HTM tissue. This study opens the way to produce biomimetic functional HTM engineered scaffolds to improve understanding of structure–function relations in the small-scale gradient or layered tissues. Permeability studies would be necessary to validate the printed models in future work.

## Supporting Information Available

Molecular weight and thermal properties of PCL before processing, after melting, and after printing; FT-IR spectra of the material; compression and tensile test results for printed scaffolds; SEM, fluorescence, and confocal images of the cells growing on the scaffolds; and exemplary image processing used for the nuclei aspect ratio quantification with ImageJ (PDF)
